# Aqueous extracts from dietary supplements influence the production of inflammatory cytokines in immortalized and primary T lymphocytes

**DOI:** 10.1186/1472-6882-9-51

**Published:** 2009-12-14

**Authors:** Paul R Hanlon, Melissa G Robbins, Chris Scholl, David M Barnes

**Affiliations:** 1 Standard Process, Department of Research and Development, 1200 West Royal Lee Dr, Palmyra, WI 53156, USA

## Abstract

**Background:**

Congaplex^® ^and Immuplex^® ^are dietary supplements that have been traditionally used to support immune system function. The purpose of these experiments was to determine whether Congaplex^® ^and Immuplex^® ^affect immune function using primary and immortalized T lymphocytes.

**Methods:**

Immortalized CEM and Jurkat T lymphocytes and primary peripheral mononuclear blood cells (PBMCs) were treated with the aqueous extracts from Congaplex^® ^and Immuplex^® ^to determine the effects of these products on cytokine production in activated T lymphocytes.

**Results:**

Congaplex^® ^enhanced phytohemagglutinin/phorbol 12-myristate 13-acetate (PHA/PMA) stimulation of both CEM and Jurkat cells as measured by the production of cytokines, while Immuplex^® ^suppressed PHA/PMA-induced production of cytokines, with the exception of interleukin (IL)-8 which was enhanced by Immuplex^®^. *In vitro *treatment of PBMCs from 10 healthy donors with Congaplex^® ^or Immuplex^® ^decreased PHA-stimulated production of interferon (IFN)-γ but increased the production of IL-13.

**Conclusions:**

While the effects of Congaplex^® ^and Immuplex^® ^differed in these two models, these data demonstrate that the aqueous extracts from these two dietary supplements can affect the inflammatory response of T lymphocytes.

## Background

CD4+ T lymphocytes protect the body from infection by bacteria, viruses and parasites [1,2]. Once activated, T helper cells in turn activate other immune cells such as macrophages, B lymphocytes and cytotoxic T lymphocytes in order to defend the individual against these infections. Activation of T lymphocytes results in production of a number of cytokines, many of which are specific for either Th1 cells, including interferon (IFN)-γ and interleukin (IL)-2, or Th2 cells, including IL-4, IL-5, IL-6, IL-10 and IL-13 [3-11].

Inflammation is associated with an increasing number of chronic diseases, and T lymphocytes are involved in the mechanisms underlying many of these [12]. Research has demonstrated that T lymphocytes are involved in a number of chronic diseases, examples of which are Crohn's disease [13], psoriasis vulgaris [14] and rheumatoid arthritis [15]. In fact, some of the most successful medications for these conditions target cytokines that are produced by T lymphocytes and prevent their action.

The context of T lymphocyte activity is crucial when interpreting whether enhancement or suppression of activity will produce a positive outcome because of the complexity of the role of immune function in infection and chronic inflammation [16,17]. In theory, optimal immune function would consist of minimal T cell activity under healthy conditions and maximal T cell activity at times of infection or under conditions where the chances of infection are increased.

Many traditionally medicines have been used to modulate immune function in an attempt to either aid an individual's ability to fight off infection [18-21] or to reduce the chance of disease produced by chronic inflammation [22-25]. Congaplex^® ^and Immuplex^® ^are products containing a complex mixture of compounds, many of which have demonstrated immunomodulatory abilities including mushrooms [26], buckwheat [27] and carrots [28]. Additionally, a number of the materials in Congaplex^® ^and Immuplex^® ^have traditionally been used to modulate immune function as part of complementary and alternative medicinal practices such as alfalfa [29], mushrooms [26] and glandular materials [30,31].

Traditionally, Congaplex^® ^and Immuplex^® ^have been used differently, with Immuplex^® ^being used under "healthy" conditions to support immune function and Congaplex^® ^being used at the onset of sickness to help support the immune system in fighting off infection. Although there is substantial anecdotal evidence to support their effectiveness, controlled *in vivo *or *in vitro *studies have not been conducted using these products. Thus, the goal of this research was to investigate the effects of aqueous extracts of Congaplex^® ^and Immuplex^® ^on the function of immortalized and primary cultured immune cells.

## Methods

### Materials

Plastic ware and general lab supplies that were used were purchased from Fisher Scientific (Hampton, NH). Chemicals and reagents, including cell culture media, were purchased from Sigma (St. Louis, MO), unless otherwise indicated. Immuplex^® ^and Congaplex^® ^were obtained from Standard Process, Inc. (Palmyra, WI).

### Cell culture

Jurkat clone E6-1 and CEM cells were obtained from ATCC (Manassas, VA) and cultured in RPMI (Sigma; St. Louis, MO) supplemented with 100 units of penicillin, 100 μg/ml of streptomycin and 10% fetal bovine serum (Atlanta Biologicals; Norcross, GA). Cultured cells were maintained at 37°C in a humidified 5% CO_2 _atmosphere. Cells were maintained between 10^5 ^and 10^6 ^cells/mL during culturing. Jurkat and CEM cells used in the experiments were between passage number 5 and 10.

### Preparation of product extracts for cell culture experiments

Immuplex^® ^and Congaplex^® ^were extracted with Jurkat growth media at a concentration of 62.5 mg/mL through gentle shaking for 20 minutes. Samples were centrifuged at 3,000 × g for 3 minutes to pellet unextracted material, and the supernatant was used to treat cells. Congaplex^® ^is a dietary supplement that contains a combination of bovine thymus extract, carrot, bovine bone, nutritional yeast, defatted wheat germ, bovine adrenal, alfalfa juice, oat flour, alfalfa flour, bovine kidney, veal bone, mushroom, buckwheat herb juice, buckwheat seed, peanut bran, soybean lecithin, soy tocopherols, carrot oil, calcium lactate, gelatin, magnesium citrate, ascorbic acid, calcium stearate and vitamin A palmitate. Immuplex^® ^is a dietary supplement that contains a combination of bovine liver, veal bone extract, nutritional yeast, bovine spleen, bovine thymus extract, ovine spleen, gelatin, zinc liver chelate, ascorbic acid, iron liver chelate, chromium yeast, copper liver chelate, selenium yeast, soy tocopherols, pyridoxine hydrochloride, calcium stearate, vitamin A palmitate, folic acid and cyanocobalamin.

### Stimulation of cultured Jurkat and CEM cells

Both Jurkat and CEM cells were stimulated using a previously described protocol [32]. Cells were plated into 24-well plates at a density of 1 × 10^6 ^cells per well in 0.5 mL of growth media. Cells were incubated for 1 hour with the extract from Immuplex^® ^and Congaplex^® ^at the indicated concentrations, and then cells were activated with phytohemagglutinin (PHA) and phorbol 12-myristate 13 acetate (PMA) at concentrations of 5 μg/mL and 7.5 nM, respectively.

### Isolation and stimulation of human PBMCs

Peripheral blood mononuclear cells (PBMCs) were obtained from five healthy male and five healthy female subjects aged 23 to 46. This research was carried out in compliance with the Helsinki Declaration as approved by an institutional review board. Informed consent was obtained from all subjects. PBMCs were isolated from heparanized blood using Accuspin Histopaque (Sigma; St. Louis, MO) according to the manufacturer's protocol. Freshly isolated PMBCs were plated into 24-well plates at a density of 5 × 10^5 ^cells per well in 0.5 mL of growth media. Cells were then incubated for 1 hour with the extract from Immuplex^® ^and Congaplex^® ^at 0.5 mg/mL, then cells were activated with 20 μg/mL of PHA. Cells were stimulated for 3 days with PHA before media was assayed for cytokine concentrations.

### Real time quantitative PCR

Four wells of cells were exposed to each treatment, as described above. For each sample, cells were transferred from the 24-well plate well into a microfuge tube and centrifuged at 1,500 × g for 5 minutes. Media was removed from the cell pellet, then cells were washed with 1 mL of PBS. Cell pellet was then centrifuged again at 1,500 × g for 5 minutes, and PBS was removed before cell pellets were stored at -80°C until RNA isolation. Total RNA was isolated per manufacturer's protocol using the MagMAX™-96 Total RNA Isolation Kit (Ambion; Austin, TX). RNA concentrations were determined via a spectrophotometer (NanoDrop; Wilmington, DE). cDNA was generated using 1.5 μg of total RNA per 50 μL cDNA reaction with the Applied Biosystems High Capacity cDNA Archive Kit, following manufacturer's protocol (Foster City, CA). Primer/probe kits for interleukin (IL)-2 (Hs99999150_m1), IL-8 (Hs99999034_m1), IL-10 (Hs99999035_m1, tumor necrosis factor (TNF)-α (Hs00174128_m1), granulocyte monocyte colony stimulating factor (GM-CSF) (Hs00171266_m1) and 18s ribosomal RNA (Hs99999901_s1) used for real time PCR quantitation of RNA were obtained from Applied Biosystems (Foster City, CA). Each treatment was represented by four replicates and each PCR reaction was carried out in duplicate. PCR was performed on an ABI 7300 Sequence Detection System. Each 25 μL reaction contained 1 μL of cDNA, 12.5 μL of 2 × Taqman Universal PCR Mastermix (Applied Biosystems; Foster City, CA) and 1.25 μL of the primer/probe reaction mixture (for the 18s ribosomal RNA reaction the primer/probe mixture diluted 1:6 before 1.25 μL was added). All reactions were run with the following parameters: 2 minutes at 50°C then 10 minutes at 95°C followed by 40 cycles of 95°C for 15 seconds and 60°C for 1 minute. Fold changes between treatment groups were then determined using the reaction efficiency [33] and 18s ribosomal RNA as the reference gene: Ratio = (E_target_)^ΔCP ^target(control - sample)/(E_ref_)^ΔCP ^ref(control - sample).

### Cell viability

Four wells of cells were exposed to each treatment, as described above. Cell viability assay was performed as per manufacturer's protocol after 24 hours of treatment (Promega MultiTox-Fluor Multiplex Cytotoxicity Assay; Madison, WI).

### Multiplex quantitation of cytokines

Four wells of cells were exposed to each treatment as described above. For each sample, cells were transferred from the 24-well plate well into a microfuge tube and centrifuged at 1,500 × g for 5 minutes. Media was removed from the cell pellet and assayed for cytokine concentrations using the Milliplex Human Cytokine Multiplex Immunoassay Kit (Millipore; Billerica, MA). Quantitation of GM-CSF, IFN-γ, IL-2, IL-4, IL-8, IL-10, IL-13 and TNF-α in all cell types was performed as per the manufacturer's protocol. Analysis was conducted on a Luminex 100 instrument (Luminex; Austin, TX) with Luminex LDS 1.7 SP2 data collection software. Calculation of cytokine concentrations was performed using cubic spline with GraphPad Prism (San Diego, CA).

### Statistical analysis

Each result is presented as mean ± standard deviation. The statistical difference between treatments was determined by analysis in GraphPad Prism (San Diego, CA) by one-way analysis of variance (ANOVA). Non-normally distributed real-time PCR data (detailed in Figure 4) were transformed using a natural log function prior to statistical analysis.

## Results

### Activation of immortalized T lymphocytes with PHA and PMA results in peak elevation of cytokine RNA levels prior to peak concentrations of cytokine concentration in the media

**Figure 1:**
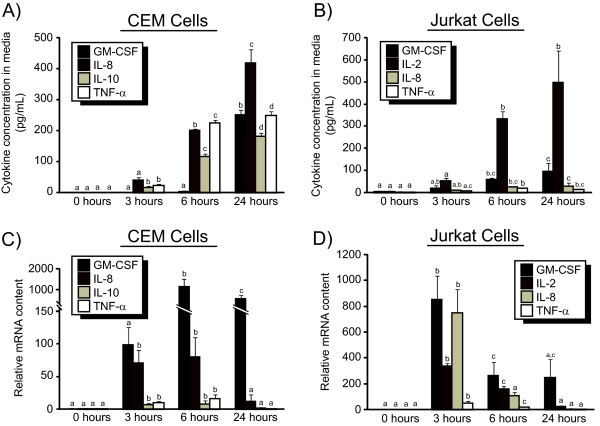
**The timecourse of mRNA concentration and cytokine release into the media over the first 24 hours after stimulation with PMA/PHA**. CEM (A, C) and Jurkat (B, D) cells were treated with PMA and PHA as described in Materials and Methods. Cells were harvested immediately before treatment (0 hour), and 3, 6 and 24 hours after treatment. Media was assayed for cytokine concentrations (A, B) using a multiplex bead assay. Cell pellets were assayed for mRNA corresponding to the same cytokines (C, D) using real-time PCR. For both cytokine and mRNA expression, data represents the average of three replicates ± standard deviation. Cytokine expression within each of the four genes (but not between genes) were compared by ANOVA with Tukey's post-hoc test. The mean concentrations of each individual cytokine were compared between each of the four timepoints. Means of groups that share a letter are not significantly different, whereas the means of any groups that do not share a letter are significantly different (p < 0.05). The concentrations of different cytokines were not compared to each other, thus display of significance only indicates differences in the concentration of the same cytokine at different timepoints. Data are representative of 3 independent experiments, using independent cell cultures, all of which produced similar results.

CEM and Jurkat T lymphocytes were activated with PHA and PMA as described in the Materials and Methods. After 0 (just prior to stimulation), 3, 6 and 24 hours following stimulation media from the cells was collected for analysis of the concentration of cytokines (Figure 1A, B) and cells were harvested for analysis of cytokine RNA (Figure 1C, D). The concentration of GM-CSF, IFN-γ, IL-2, IL-4, IL-8, IL-10, IL-13 and TNF-α in the media of both Jurkat and CEM cells was measured, and the results from the four most abundant cytokines are shown in Figures 1A and 1B. The concentrations of the cytokines in the media that are not shown were all below 100 pg/mL at the 24 hour timepoint (data not shown). In both cell lines the peak concentrations were determined to be 24 hours after stimulation for all of the cytokines measured. In comparison, RNA was maximally expressed 6 hours and 3 hours after stimulation for all four cytokines measured for CEM and Jurkat cells, respectively.

### Congaplex^® ^and Immuplex^® ^extracts affect PHA/PMA-mediated cytokine production in immortalized T lymphocytes in opposing directions

**Figure 2:**
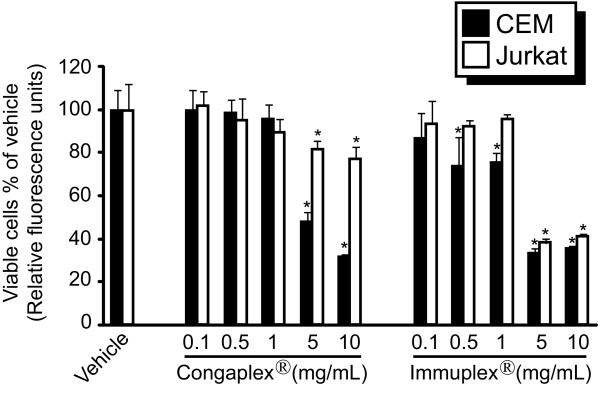
**Congaplex^® ^and Immuplex^® ^decrease the number of viable CEM and Jurkat cells at concentrations of 5 and 10 mg/mL**. CEM and Jurkat cells were treated with Congaplex^® ^and Immuplex^® ^at the indicated concentrations for 1 hour prior to the addition of PMA and PHA. After 24 hours of treatment with PMA and PHA the number of viable cells was measured as described in Materials and Methods. Data were normalized to the relative fluorescence units for the vehicle treatment of both CEM (black bars) and Jurkat (white bars) cells. Data represents the average of four replicates ± standard deviation. * = significantly different from the vehicle treatment. Data are representative of 3 independent experiments, using independent cell cultures, all of which produced similar results.

Congaplex^® ^and Immuplex^® ^extracts, prepared as described in Materials and Methods, were applied to cultures of CEM and Jurkat T lymphocytes and cells were incubated with the cells for 1 hour prior to stimulation with PHA and PMA. Cells were treated with Congaplex^® ^and Immuplex^® ^extracts at 0.1, 0.5, 1, 5 and 10 mg/mL and the viability of the cells was assessed 24 hours after stimulation (Figure 2). Congaplex^® ^and Immuplex^® ^significantly reduced the viability of both cell lines at 5 and 10 mg/mL, and Congaplex^® ^did not significantly affect cell viability in either cell line at the 1 mg/mL concentration or lower. Immuplex^® ^did not reduce Jurkat cell viability at concentrations up to 1 mg/mL, however, there was significant reduction in CEM viability at 0.5 and 1 mg/mL.

**Figure 3:**
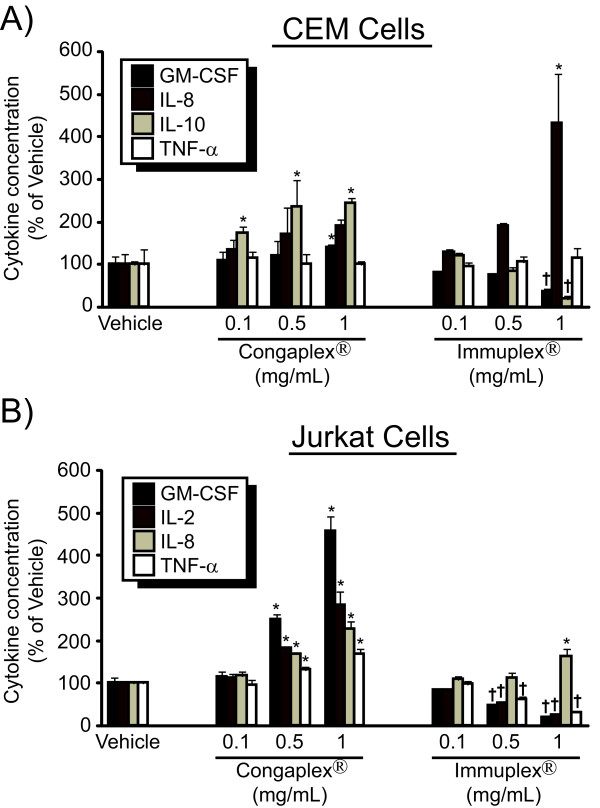
**The aqueous extract from Immuplex^® ^and Congaplex^® ^alter cytokine release following activation**. CEM (A) and Jurkat (B) cells were pre-treated the aqueous extract from Immuplex^® ^or Congaplex^® ^for one hour prior to treatment with PMA and PHA as described in Materials and Methods. Cells were harvested 24 hours after PMA/PHA treatment and media was assayed for cytokine concentrations using a multiplex bead assay. Data are representative of 3 independent experiments, using independent cell cultures, all of which produced similar results. Data represents the average of four replicates ± standard deviation. Data is presented as the percentage of cytokine concentration in cells treated with PMA and PHA in the absence of Immuplex^® ^or Congaplex^® ^treatment (vehicle). * = significantly higher than vehicle treatment. † = significantly lower than vehicle treatment.

**Figure 4:**
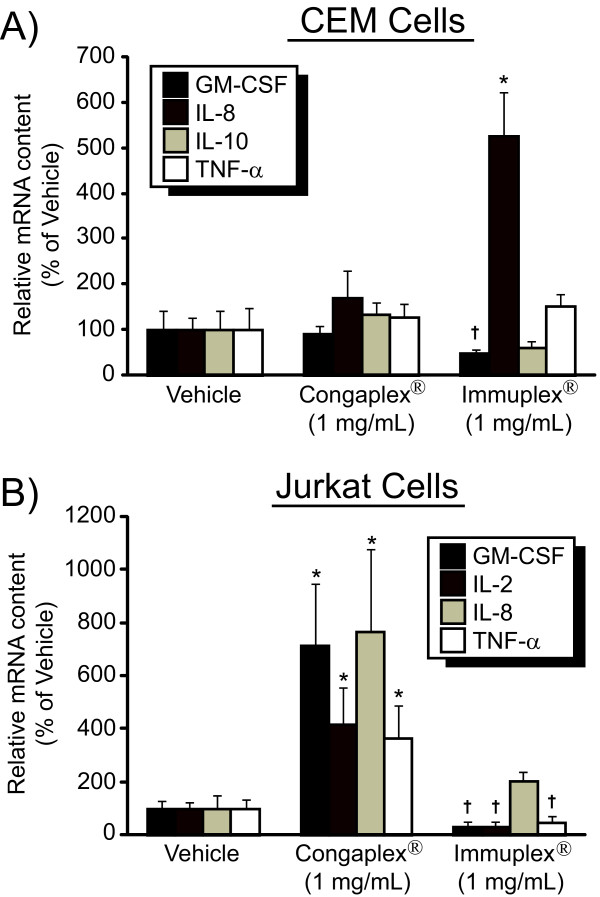
**The aqueous extract from Immuplex^® ^and Congaplex^® ^alter cytokine RNA expression following activation**. CEM (A) and Jurkat (B) cells were pre-treated the aqueous extract from Immuplex^® ^or Congaplex^® ^for one hour prior to treatment with PMA and PHA as described in Materials and Methods. CEM and Jurkat cells were harvested 6 and 3 hours after PMA/PHA treatment, respectively, and cells were assayed for mRNA expression corresponding to cytokines using real-time PCR. Data are representative of 3 independent experiments, using independent cell cultures, all of which produced similar results. Data represents the average of four replicates ± standard deviation. Data is presented as the percentage of cytokine concentration in cells treated with PMA and PHA in the absence of Immuplex^® ^or Congaplex^® ^treatment (vehicle). * = significantly higher than vehicle treatment. † = significantly lower than vehicle treatment.

The concentration of cytokines in the media was then measured 24 hours after PMA and PHA stimulation, when the concentration of cytokines in the media was at maximum levels (Figure 1A, B). Treatment of CEM cells with Congaplex^® ^resulted in a significant increase in IL-8 and IL-10 concentrations in the media, while treatment of these cells with Immuplex^® ^resulted in a significant increase in IL-8 and significant decreases in the concentration of GM-CSF and IL-10 (Figure 3A). In CEM cells, TNF-α concentration in the media was not significantly affected by either Congaplex^® ^or Immuplex^®^. Incubation of Jurkat cells with Immuplex^® ^resulted in a dose-dependent increase in the concentration of IL-8 and a dose-dependent decrease in the concentration of GM-CSF, IL-2 and TNF-α (Figure 3A). Incubation of Jurkat cells with Congaplex^® ^resulted in a dose-dependent increase in GM-CSF, IL-2, IL-8 and TNF-α (Figure 3B). In the absence of PMA/PHA stimulation Congaplex^® ^and Immuplex^® ^did not have significant effects on cytokine production (data not shown). RNA was harvested from CEM and Jurkat T lymphocytes after 6 and 3 hours of stimulation with PHA and PMA, respectively, to achieve maximum expression (Figure 1C, D). The effects of treatment with the 1 mg/mL concentration of Immuplex^® ^and Congaplex^® ^on RNA expression showed the same trend as seen in the cytokine concentration in the media of both CEM and Jurkat cells (Figure 4A, B).

### Congaplex^® ^and Immuplex^® ^modulate cytokine release in PMBCs isolated from 10 different donors

**Figure 5:**
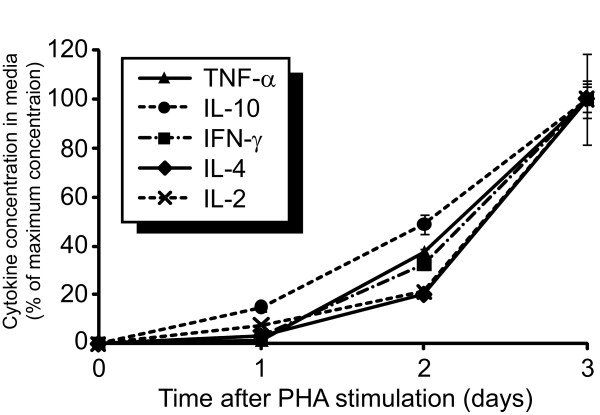
**The timecourse of cytokine concentrations into the media from PBMCs over the first 3 days after stimulation with PHA**. PBMCs from a healthy volunteer were exposed to PHA as described in Materials and Methods. Media from the cells was collected 1, 2 and 3 days after stimulation and was assayed for cytokine concentrations using a multiplex bead assay. Data is presented as the percentage of cytokine concentration in cells at the timepoint with maximum cytokine concentration, which in each case was 3 days after PHA stimulation. Data represents the average of three replicates ± standard deviation.

Prior to analysis of cytokine expression from multiple donors, PBMCs from a single donor were stimulated with PHA and then 1, 2 and 3 days following stimulation media from the cells was collected for analysis of the concentration of several cytokines (Figure 5). The media concentration of TNF-α, IL-2, IL-4, IL-10 and IFN-γ increased on each day, reaching maximum concentrations of each cytokine three days following stimulation.

**Figure 6:**
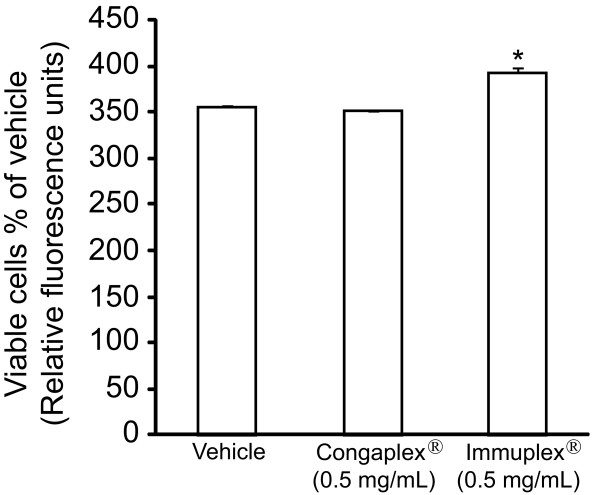
**Congaplex^® ^and Immuplex^® ^do not decrease the number of viable PBMCs at 0.5 mg/mL**. PBMCs from a healthy volunteer were treated with Congaplex^® ^and Immuplex^® ^at 0.5 mg/mL for 1 hour prior to the addition of PHA. After 24 hours of treatment with PHA the number of viable cells was measured as described in Materials and Methods. Data were normalized to the relative fluorescence units for the vehicle treatment. Data represents the average of three replicates ± standard deviation. * = significantly different from the vehicle treatment.

**Figure 7:**
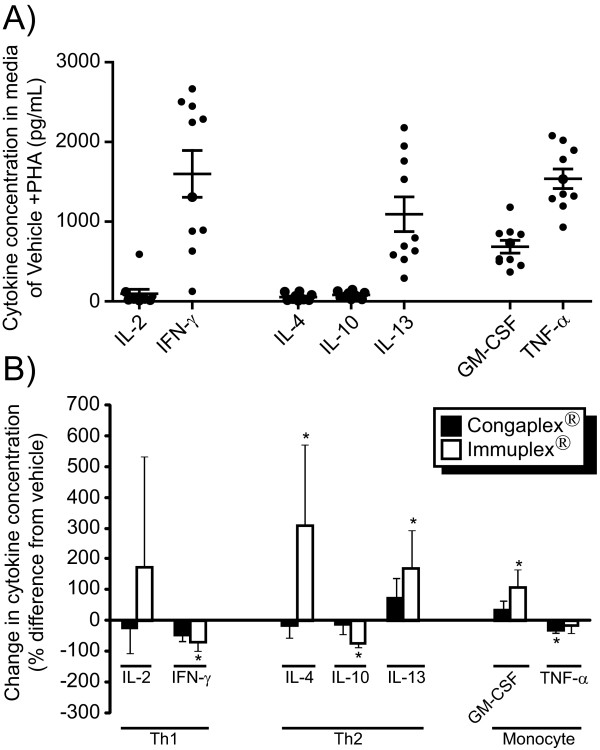
**The aqueous extract from Immuplex^® ^and Congaplex^® ^reduce cytokine release by human PBMCs in a dose-dependent manner**. PBMCs from 10 healthy volunteers were pre-treated with the aqueous extract from Immuplex^® ^or Congaplex^® ^at 0.5 mg/mL for one hour prior to treatment with PHA as described in Materials and Methods. Cells were harvested 3 days after treatment and media was assayed for cytokine concentrations using a multiplex bead assay. A) A scatter plot shows the concentration of each cytokine in the media from each of the 10 donors after treatment with PHA, in the absence of Congaplex^® ^or Immuplex^®^. The average of all 10 donors is indicated as a horizontal bar ± standard deviation. For each donor, data represents the average of three replicates ± standard deviation. B) The effects of Congaplex^® ^and Immuplex^® ^on cytokine concentrations in the media of cells treated with PHA. Data are expressed as the percentage difference from treatment with PHA and vehicle treatment. The data are presented as the average response of the PBMCs from the 10 donors ± standard deviation. * = significantly different from the vehicle treatment.

PBMCs were isolated from 10 donors as described in the Materials and Methods, then cells were treated with Congaplex^® ^and Immuplex^® ^(0.5 mg/mL) for 1 hour before being stimulated with PHA. Neither Congaplex^® ^nor Immuplex^® ^reduced the number of viable PBMCs at this concentration (Figure 6). The same cytokines that were measured in the immortalized cells (GM-CSF, IFN-γ, IL-2, IL-4, IL-8, IL-10, IL-13 and TNF-α), were measured in the media 3 days after stimulation (Figure 7). The media from unstimulated PBMC cells had cytokine concentrations below the level of detection with the exception of IL-8, which had an average media concentration of 1262.2 pg/mL ± 1089.0. The concentration of the other seven cytokines in the treatment media varied between donors after 3 days of PHA stimulation (Table 1, Figure 7A), and of these cytokines only four (IFN-γ, IL-13, GM-CSF and TNF-α) had average media concentrations of greater than 100 pg/mL. Immuplex^® ^significantly decreased expression of IFN-γ and significantly increased IL-13 (Figure 7B), the two most abundant T lymphocyte-specific cytokines. Congaplex^® ^trended to have a similar effect as Immuplex^® ^on the concentration of these two cytokines, although the differences were not significant.

**Table 1:** Cytokine concentration in PHA-stimulated PBMCs on day 3 of culture

	Range (pg/mL)	Avg ± StDev (pg/mL)
IL-2	10 - 592	96 ± 177
IFN-γ	127 - 2667	1600 ± 929
IL-4	8 - 127	53 ± 44
IL-10	26 - 145	81 ± 41
IL-13	290 - 2178	1094 ± 686
GM-CSF	368 - 1181	685 ± 252
TNF-α	932 - 2078	1539 ± 386

## Discussion

**Figure 8:**
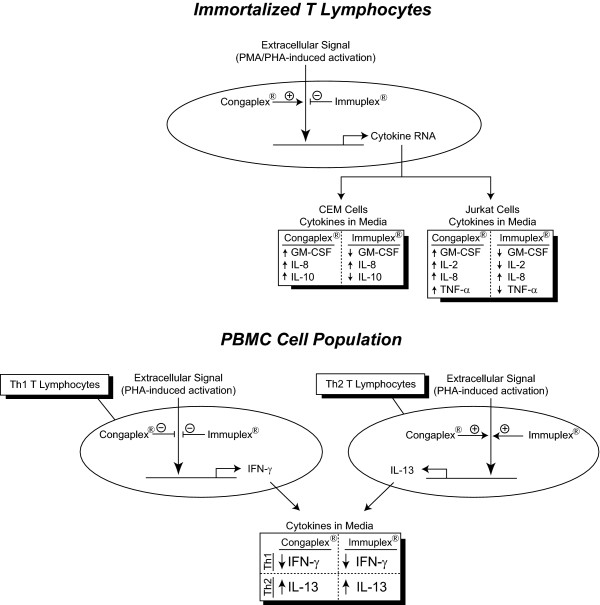
**Hypothetical diagram of the mechanism through which Congaplex^® ^and Immuplex^® ^affect T lymphocyte function**. *Top *Congaplex^® ^and Immuplex^® ^regulated the PHA/PMA-induced expression of RNA corresponding to cytokines resulting in an increase or decrease, respectively, of cytokine concentrations in the media *Bottom *Congaplex^® ^and Immuplex^® ^decreased the production of IFN-γ, the predominant Th1-specific cytokine detected in the media, and increased the production of IL-13, the predominant Th2-specific cytokine present in the media, in response to PHA.

The results of these experiments demonstrate that the aqueous extract from Congaplex^® ^and Immuplex^® ^had immunomodulatory effects in both immortalized cells (CEM and Jurkat) as well as cultures of human primary blood mononuclear cells (PBMCs). Congaplex^® ^and Immuplex^® ^had opposing effects on PMA/PHA-induced activation of CEM and Jurkat cells, with Congaplex^® ^enhancing the stimulation and Immuplex^® ^inhibiting the stimulation (Figure 8). Alternatively, in the PBMCs the effects were similar with both products, inhibiting production of the most abundant Th1-related cytokine (IFN-γ) and stimulating production of the most abundant Th2-related cytokine (IL-13).

The results from the immortalized cell lines support the use of Congaplex^® ^under conditions of infection or increased chance of infection as a way to enhance the T lymphocyte response that will protect the body from infections, while Immuplex^® ^would be used under healthy conditions to suppress T lymphocyte-mediated inflammation that increases the risk of diseases such as Crohn's disease, psoriasis vulgaris and rheumatoid arthritis. Alternatively, the results from the PBMC experiments suggest that Congaplex^® ^and Immuplex^® ^may both enhance the Th2 response while suppressing the Th1 response. Other natural products have been demonstrated to have immunomodulatory effects, some of which enhance T lymphocyte activation [34-36] while some suppress activation [37-39]. Congaplex^® ^and Immuplex^® ^may be working through the same or different mechanisms than these other natural products, and therefore optimal *in vivo *activity could be accomplished through alternative therapies that combine two or more of these supplements that have demonstrated immunomodulatory properties.

These data have also demonstrated that the effects of Congaplex^® ^and Immuplex^® ^were manifested at the level of gene expression, as demonstrated by changes in both cytokine levels (Figure 3) in the media as well as RNA levels (Figure 4). Additionally, since the aqueous extract from these supplements did not affect cytokine production in the absence of the PMA/PHA stimulus, these supplements are not working through a mechanism of direct activation of these cells. This demonstrates that in the immortalized CEM and Jurkat cells Congaplex^® ^and Immuplex^® ^are modulating the transcription of inflammatory proteins.

The systematic increase and decrease in the expression of inflammatory cytokines is critical for the control of the inflammatory response [40,41]. Figure 1C and 1D demonstrate a rapid (within 3 hours) upregulation of cytokine RNA followed by a decline in mRNA expression levels by 24 hours. These data do not provide information about the mechanism through which mRNA levels for these cytokines are decreasing, but possible mechanisms include decreases in transcription, transcriptional repression and/or a decrease in mRNA stability [42].

The differences of the response of immortalized cells and PBMCs to Congaplex^® ^and Immuplex^® ^are likely due to the differences between these experimental models. Immortalized cells such as the CEM and Jurkat cell lines have been used extensively to study T cell function; however, these cells are more closely related to leukemia cells than healthy T lymphocytes, and therefore the relevance of these results to the activity of healthy T lymphocytes may be limited. Additionally, immortalized cells are a simplified model since cultures of these cells consist of only one cell type, whereas PBMCs are a mixture of multiple cell types. Therefore, interactions between cells in the response of immune cells to Congaplex^® ^and Immuplex^® ^would be overlooked in experiments using either CEM or Jurkat cells.

However, the results from the immortalized CEM and Jurkat cells can have relevance to *in vivo *T lymphocyte function. Immortalized T lymphocytes have been used to predict the *in vivo *immunomodulatory activity of many natural products [43,44], including investigating the mechanisms of T lymphocyte function [45,46]. The results presented here show that the effect of Congaplex^® ^and Immuplex^® ^are similar in two different T lymphocyte cells lines (CEM and Jurkat). This strengthens the possibility that the results of these experiments are due to generalized effects these extracts have on T lymphocytes rather than effects to the specific immortalized cell line. Furthermore, while the comparison of these *in vitro *experiments (with both immortalized and primary lymphocytes) suggest that there may be complicated interactions occurring *in vivo*, it also demonstrates the importance of using more than one model to try to understand effects on materials on a system as complex as the immune system.

Another source of complication in this experimental model is the fact these dietary supplements are mixtures of a number of different raw materials, and many of the raw materials (such as alfalfa juice or buckwheat seed powder) are also mixtures of countless nutrients and other phytochemicals. This complexity of the materials, unlike what is the case with pharmaceuticals or isolated phytochemicals, is much more likely to have effects through multiple mechanisms. This is also demonstrated by the fact that foods, which are complex mixtures, have been shown to affect immunity through a number of mechanisms [16].

Furthermore, the extracts used in these experiments certainly underestimate the complexity of what an individual will be exposed to when taking these supplements orally. The bioavailability of the compounds in these supplements, which will be affected by many things including the digestive process, will be much different *in vivo *than what the immune cells were exposed to in terms of an aqueous extract in these experiments. Additionally, while the extracts used in these experiments are likely to represent most of the polar compounds present in these supplements, the non-polar compounds in the supplements were not likely to be extracted. The non-polar compounds in the supplements could directly modulate immune cell function or work to affect how the polar compounds affect immune cell function, however the experiments described here do not address this possibility.

While these initial experiments have demonstrated that Immuplex^® ^and Congaplex^® ^can have immunomodulatory effects *in vitro*, *in vivo *studies would be required to determine what effects these products would have in the context of the complexity of the whole organism where issues, including bioavailability and cell-cell interactions, could have important consequences. Furthermore, the data from our ten donors also suggests that while the response to Immuplex^® ^and Congaplex^® ^may differ significantly between individuals. Certainly genetic differences and differences between the abundance of different cell types could have a large impact on how an individual might respond to a supplement. Whether or not experiments with PBMCs could be predictive of an individual's response to these supplements would also be of interest.

## Conclusions

Many traditional medicines are used in an attempt to modulate immune function, however little evidence exists supporting the activity of many of these medicines. This report presents initial *in vitro *data supporting the anecdotal evidence of the efficacy of Congaplex^® ^and Immuplex^® ^in affecting immune function. The preliminary data presented here demonstrating that Congaplex^® ^and Immuplex^® ^can have immunomodulatory effects *in vitro *provides additional justification to conduct subsequent animal and human trials that further investigate the activity of these supplements.

## Competing interests

The authors of this publication are all employees of Standard Process, Inc., manufacturer of Immuplex^® ^and Congaplex^®^.

## Authors' contributions

PH participated in the design of the study, cultured both the immortalized and primary cells and drafted the manuscript. MR and CS conducted the measurements of cytokine and RNA and contributed to portions of the manuscript. DB participated in the design of the study and contributed to the manuscript. All authors read and approved the final manuscript.

## Pre-publication history

The pre-publication history for this paper can be accessed here:

http://www.biomedcentral.com/1472-6882/9/51/prepub
